# Genotype‐by‐environment interaction informs selection for seed physiological quality and stink bug tolerance in soybean under pest management contrasts

**DOI:** 10.1002/ps.70904

**Published:** 2026-05-11

**Authors:** Larissa Chamma, Rômulo Pedro Macêdo Lima, Gustavo Ferreira da Silva, Adriano Abreu Moreira, Thiago Barbosa Batista, Samara Moreira Perissato, José Baldin Pinheiro, Edvaldo Aparecido Amaral da Silva

**Affiliations:** ^1^ Department of Crop Production Faculty of Agronomic Sciences, São Paulo State University (UNESP) Botucatu Brazil; ^2^ Department of Biotechnology and Plant and Animal Production Federal University of São Carlos Araras Brazil; ^3^ Luiz de Queiroz School of Agriculture/University of São Paulo Department of Genetics Piracicaba Brazil; ^4^ University of São Paulo, Center for Nuclear Energy in Agriculture Piracicaba Brazil; ^5^ Federal University of Paraná Department of Agronomic Sciences Palotina Brazil

**Keywords:** soybean, stink bugs, host plant tolerance, integrated pest management, seed longevity, genotype‐by‐environment interaction

## Abstract

**BACKGROUND:**

Stink bugs are among the most economically important pest complexes in soybean, causing direct injury to developing seeds, reducing physiological quality, and increasing reliance on insecticide‐based control. Although host plant tolerance is a central component of integrated pest management (IPM), its contribution to stabilizing seed physiological performance under field infestation remains insufficiently characterized within a genotype‐by‐environment interaction. We evaluated two contrasting parental lines and 12 recombinant inbred lines (RILs) across two soybean‐growing regions in Brazil under contrasting management scenarios (with and without insecticide application). Eight seed physiological traits related to germination, vigor, and longevity were assessed to determine the stability of host plant responses under stink bug exposure.

**RESULTS:**

Environmental effects predominated for early vigor traits, whereas seed longevity showed comparatively stronger genetic control. RILs 109, 186, and 227, together with the tolerant parent IAC‐100, maintained superior seed physiological performance under infestation, including in the absence of insecticide application. The Anhumas without insecticide‐based control environment provided the highest discriminatory power for identifying tolerant materials. Moderate to strong positive correlations among physiological traits suggest that longevity can serve as a robust decision trait to anticipate broader seed performance under pest stress.

**CONCLUSION:**

These findings demonstrate that host plant tolerance can mitigate stink bug‐induced losses in seed quality and support the integration of tolerant cultivars into sustainable pest management strategies, potentially reducing dependence on insecticides while enhancing production stability. This supports cultivar deployment as a proactive IPM tactic to buffer seed quality losses when chemical control is limited or inconsistent. © 2026 The Author(s). *Pest Management Science* published by John Wiley & Sons Ltd on behalf of Society of Chemical Industry.

## Graphical abstract

Genotype‐by‐environment interaction shaped soybean seed stability under contrasting insecticide regimes. Seed longevity emerged as a stable, management‐relevant trait under pest pressure. High‐pressure, no‐insecticide conditions improved discrimination of tolerant genotypes for integrated pest management deployment.

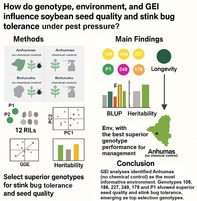

## 1 INTRODUCTION

Soybean production worldwide is strongly constrained by insect pests capable of compromising yield and seed marketability. Among these, stink bugs are one of the most economically important pest complexes, causing direct injury to pods and developing seeds, and reducing both productivity and seed quality.^1^ Feeding damage can impair the seed‐to‐seedling transition, induce necrosis, and reduce germination, generating losses that extend beyond field productivity into crop establishment.

Seed quality reflects the genetic potential of a genotype to respond to biotic and abiotic factors within the production environment.^2^ Climatic and nutritional stresses during reproductive development, together with insect and microbial damage, alter seed physiological attributes in a genotype‐dependent manner.^3^, ^4^, ^5^ In soybean, stink bug injury represents a major biotic driver of these changes, reinforcing the need for management strategies capable of preserving seed physiological integrity.

Stink bug management includes chemical, biological, and genetic approaches.^6^, ^7^ Although insecticide spraying remains widely adopted and provides residual protection,^8^, ^9^ integrated strategies combining chemical and non‐chemical tactics are generally more effective than isolated methods.^9^ Within integrated pest management (IPM) frameworks, host plant tolerance represents a sustainable alternative because it reduces dependence on insecticides while maintaining crop performance. Breeding programs therefore play a central role in developing cultivars that combine agronomic performance with tolerance to stink bug attack and high physiological seed quality.^10^, ^11^

Seed quality traits are shaped by interactions between genetic background and environmental conditions, making genotype‐by‐environment interaction (GEI) analysis essential for identifying stable and adaptable genotypes.^12^, ^13^, ^14^ Multi‐environment trials and statistical approaches such as joint analysis of variance (ANOVA) and regression models have been widely used to partition genotype, environment, and GEI effects.^15^, ^16^, ^17^ However, GEI studies focusing specifically on seed physiological traits under stink bug infestation levels remain scarce because most research has emphasized yield‐related traits. This gap limits understanding of the stability of seed quality under biotic stress.

This study evaluated GEI effects on soybean genotypes derived from two contrasting parental lines (IAC 100 and CODETEC 215) and their recombinant inbred lines under natural stink bug infestation levels across multiple production scenarios. Rather than focusing solely on breeding performance, we aimed to determine how host plant tolerance contributes to maintaining seed physiological quality under contrasting pest management conditions. By integrating multi‐environment analysis with realistic infestation scenarios, this study provides applied evidence to support host plant tolerance as a functional component of IPM in soybean systems.

## 2 MATERIAL AND METHODS

### 2.1 Plant material

The soybean population consisted of 229 recombinant inbred lines (RILs) derived from the cross between IAC 100 (tolerant to the stink bug complex) and CODETEC 215 (susceptible), developed by the Soybean Breeding Program at ESALQ/USP.^18^ At the time of evaluation, the population was at the F14 generation. From this population, 12 RILs were selected based on variability in seed physiological quality and tolerance to the stink bug complex, together with the two parental lines, totaling 14 genotypes evaluated in this study (Table 1).

**Table 1:** Preliminary classification of genotypes based on seed physiological quality and tolerance to stink bug complex^18^

Genotypes	Groups
IAC‐100 e CD‐215 (Group 1)	Parents
R‐109, R‐186 e R‐227 (Group 2)	High seed physiological quality and tolerance to stink bug complex
R‐007, R‐241 e R‐249 (Group 3)	High seed physiological quality and susceptibility to stink bug complex
R‐119, R‐144 e R‐179 (Group 4)	Low seed physiological quality and tolerance to stink bug complex
R‐036, R‐113 e R‐147 (Group 5)	Low seed physiological quality and susceptibility to stink bug complex

### 2.2 Production area and experimental design

The experiment was conducted in two experimental areas in São Paulo State, Brazil: (i) Fazenda Lageado, Faculty of Agronomic Sciences, São Paulo State University (UNESP – Botucatu), and (ii) the Experimental Station of the Department of Genetics and Plant Breeding at ESALQ/USP, in Anhumas (Piracicaba municipality).

Prior to sowing, liming and mineral fertilization were performed according to soil analysis recommendations. Soil preparation included leveling, and sowing was carried out mechanically. Seeds assigned to chemical treatment were previously treated according to manufacturer recommendations with fungicide (carboxin + thiram), insecticide (thiamethoxam), and inoculant containing *Bradyrhizobium* sp. Weed control was conducted using herbicide (haloxyfop‐R‐methyl) followed by manual weeding when necessary. Preventive fungicide applications were performed at regular intervals throughout the crop cycle.

Insecticide applications were conducted based on pest monitoring. In plots designated for insecticide‐based control of stink bugs, insecticides were applied weekly or whenever the beat cloth method detected at least one stink bug per square meter. The insecticides comprised thiamethoxam + lambda‐cyhalothrin (0.028 + 0.21 kg a.i. ha^−1^), chlorantraniliprole + bifenthrin (0.024 + 0.078 kg a.i. ha^−1^), imidacloprid + beta‐cyfluthrin (0.05 + 0.00625 kg a.i. ha^−1^), deltamethrin (0.0075 kg a.i. ha^−1^), and imidacloprid + bifenthrin (0.05 + 0.01 kg a.i. ha^−1^). Monitoring was performed from flowering to full maturity.^19^ In the non‐insecticide treatment, no insecticides were applied; however, all other agronomic practices (fertilization, fungicides, and weed management) were maintained equally across treatments. An insecticide mode‐of‐action rotation strategy was adopted to minimize the development of resistance and maintain control efficacy.

For GEI analyses, each location‐by‐management combination was considered a distinct evaluation environment. Thus, a 2 × 2 factorial design was established, combining two regions (Botucatu and Anhumas) and two stink bug management strategies (with or without insecticide‐based control), resulting in four evaluation environments: AC (Anhumas with insecticide‐based control), AP (Anhumas without insecticide‐based control), BC (Botucatu with insecticide‐based control), and BP (Botucatu without insecticide‐based control). The experiment followed a randomized complete block design with four replicates per genotype. Plots consisted of four rows spaced 0.45 m apart, with the two central rows used as the harvest area. The crop cycle extended from November 2019 to March 2020 in Botucatu and from December 2019 to March 2020 in Anhumas.

Both regions are classified as CWa climate according to Köppen, characterized by mesothermal conditions with dry winters and mean annual rainfall of approximately 1400 mm.^20^ Soils are classified as dystrophic Red Nitisol (Botucatu) and dystrophic Yellow Latosol (Anhumas).^21^

During seed production, stink bug populations were monitored throughout the reproductive stages. Harvest was performed manually, followed by mechanical threshing. Seeds were cleaned to remove malformed or visibly damaged units and stored at 20 °C and 70% relative humidity until physiological evaluations were performed at the Seed Laboratory of UNESP (Botucatu).

### 2.3 Measurement of eight variables related to seed quality

For the germination test, four replicates of 25 seeds per genotype were used (initial moisture content 9.6%). Seeds were placed on paper towel sheets moistened with distilled water at 2.5 times the dry paper weight, rolled, and maintained in sealed plastic bags in a germination chamber at 25 °C in the dark. Evaluations were conducted on the fifth day (first germination count, PC) and eighth day (final germination, G), according to the Rules for Seed Analysis,^22^ considering only normal seedlings.

Mean time to 50% germination (T50) was determined using four replicates of 25 seeds per genotype placed in Petri dishes with moistened paper towels (2.5× dry paper weight) and incubated at 25 °C in the dark. Germinated seeds (radicle protrusion >2 mm) were recorded every 6 h. T50 values were calculated from cumulative germination data using POMONA software^23^ and expressed in hours.

Seedling length was evaluated using four replicates of 10 seeds per genotype. Seeds were positioned on moistened germination paper (2.5× dry weight) with the micropyle oriented downward to ensure uniform growth, following the seed vigor testing procedure described by Nakagawa,^24^ and incubated vertically at 25 °C in the dark for 7 days. Mean shoot length (MSPA) and primary root length (MSR) were measured in centimeters. Subsequently, shoot and root tissues were separated, dried at 60 °C to constant weight, and weighed to obtain shoot dry mass (CPA) and root dry mass (CR).

Seed longevity was assessed following Zinsmeister *et al*.^25^ Seeds were stored in hermetic containers over saturated sodium chloride solution (75% RH) at 35 °C. At predetermined intervals, four replicates of 25 seeds per genotype were evaluated for viability based on radicle protrusion, as described for T50. Longevity curves were fitted using the Ellis and Roberts^26^ viability equation expressed in probit units:

$$\text{probit}\left(v\right)=K_{i}-\frac{p}{\sigma }$$

where *probit(v)* represents seed viability at storage time *p* (days), expressed in probit units, *K*_*n*_ (Ki) is the initial viability in probit units (intercept of the regression), *p* is the storage period (days), and *σ* is the longevity parameter (days), which is inversely related to the slope of the regression line (slope = −1/σ).

Seed longevity (p50) was estimated as the time required for viability to decline to 50%, corresponding to probit(v) = 0, and calculated as:

$$p_{50}=K_{i}\sigma$$

For p50, a one‐way ANOVA was performed independently within each environment (AC, AP, BC, BP), followed by Fisher's LSD test using the agricolae package in R.^27^ Means ± standard error were presented in bar plots, and significant differences were indicated by distinct letters.

### 2.4 Statistical and GEI analyses

#### 2.4.1 General descriptive statistics

Because stink bug exposure varied among environments, GEI analyses were performed to identify genotypes able to maintain seed physiological quality under contrasting pest management conditions. Descriptive statistics and exploratory analyses (boxplots, histograms, and scatter plots) were conducted using the *metan* package in R.^28^ Heatmaps were generated to visualize genotype performance across the four evaluation environments for each trait.

#### 2.4.2 Joint ANOVA

A joint ANOVA was performed to evaluate the effects of genotype (GEN), environment (ENV), and their interaction (GEN × ENV) on the eight seed physiological traits. The linear model adopted was:

$$Y_{\mathrm{ij}}=\mu +\alpha_{i}+\tau_{j}+{\left(\alpha \tau \right)}_{\mathrm{ij}}+\varepsilon_{\mathrm{ij}}$$

where $Y_{\mathrm{ij}}$ represents the response of genotype *i* in environment *j*, μis the overall mean, $\alpha_{i}$is the fixed effect of genotype, $\tau_{j}$is the random effect of environment, 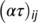
is the interaction effect, and $\varepsilon_{\mathrm{ij}}$is the residual error. Homogeneity of residual variances was verified prior to combined analysis. The significance of main and interaction effects was assessed at *P* < 0.05.

#### 2.4.3 Correlation analysis

Pearson correlation coefficients among the eight physiological traits were estimated using the *metan* package in R.^28^ Correlation matrices were visualized as heatmaps to explore the strength and direction of associations among germination, vigor, and longevity traits.

#### 2.4.4 AMMI biplot model

The additive main effects and multiplicative interaction (AMMI) model was applied to investigate GEI structure.^28^ The model combines ANOVA for additive effects with principal component analysis (PCA) of the interaction matrix:

$$Y_{\mathrm{ij}}=\text{𝜇}+{\text{𝑔}}_{\text{𝑖}}+{\text{𝑎}}_{\text{𝑗}}+\sum_{k=1}^{n}\lambda_{k}\gamma_{\mathrm{ik}}\;\alpha_{\mathrm{jk}}+{\text{𝑟}}_{\text{𝑖𝑗}}+{\text{𝜀}}_{\text{𝑖𝑗}}$$

where $g_{i}$ and $a_{j}$represent genotype and environment effects, respectively, $\lambda_{k}$ is the singular value for the *k*‐th interaction principal component axis (IPCA), $\gamma_{\mathrm{ik}}$and $\alpha_{\mathrm{jk}}$are genotype and environment scores, and $r_{\mathrm{ij}}$represents residual interaction. AMMI1 and AMMI2 biplots were generated to evaluate genotype stability and adaptability. Genotypes with lower absolute interaction scores were considered more stable across environments. For the AMMI analysis of p50, genotype and environment scores on the first two interaction principal components (PC1 and PC2) were extracted to support biplot interpretation.

#### 2.4.5 GGE biplot model

The genotype plus genotype‐by‐environment (GGE) biplot approach was used to identify mega‐environments and evaluate genotype performance and stability.^29^, ^30^, ^31^ The model decomposes environment‐centered data using singular value decomposition:

$${\text{𝑌}}_{\text{𝑖𝑗}}–\text{𝜇}-{\text{𝐸}}_{\text{𝑗}}=\sum_{k=1}^{2}\lambda_{k}\xi_{\mathrm{ik}}\;\eta_{\mathrm{jk}}+{\text{𝜀}}_{\mathrm{ij}}$$

where $E_{j}$represents the environmental main effect, and $\lambda_{k}$, $\xi_{\mathrm{ik}}$, and $\eta_{\mathrm{jk}}$correspond to singular values and principal component scores for genotypes and environments. The ‘which‐won‐where,’ ideal genotype, and ideal environment views were generated to support selection decisions.

#### 2.4.6 BLUP and genetic parameter estimation

Mixed linear models were fitted using restricted maximum likelihood (REML), considering genotype and GEI as random effects. The general model adopted was:

y=Xβ+Zu+ε

where y is the vector of observations, βrepresents fixed effects, urepresents random effects (genotype and GEI), and εrepresents the residuals.

Best linear unbiased predictions (BLUPs) were obtained for genotype performance across environments. Stability was assessed using the weighted average of absolute scores from BLUP interaction (WAASB).^32^ The likelihood ratio test was applied to assess the significance (*P* < 0.05) of random effects. The estimated genetic parameters were *H* ^2^, the broad‐sense heritability, calculated as *H* ^2^ = Vg/(Vg + VGEI + VR), where Vg is the genotypic variance, VGEI is the GEI variance, and VR is the residual variance, R^2^GEI is the coefficient of determination for GEI effect, calculated as R^2^GEI = VGEI/(Vg + VGEI + VR), Ac is the selection accuracy, estimated as Ac = $\sqrt{H2}$, and *r*_ge_ is the genotype‐by‐environment correlation, estimated as *r*_ge_ = V_g_/(V_g_ + V_GEI_). All BLUP model analyses were conducted using the *metan* package in R software.^28^

#### 2.4.7 MTSI model

The Multi‐Trait Stability Index (MTSI) was applied to rank genotypes based on simultaneous mean performance and stability across environments.^32^ The index is calculated as:

$${\text{MTSI}}_{i}={\left[\sum_{j=1}^{f}\left(\mathrm{\gamma ij}-\mathrm{\gamma j}\right)2\right]}^{0.5}$$

where *γ*_*ij*_ represents the score of genotype *i* for factor *j*, and $\gamma_{j}$corresponds to the ideal genotype.

Genotypes were selected based on predefined selection intensity, and predicted selection differentials were calculated for all traits, as well as the identification of TOP1 (best‐performing genotype) in each evaluated environment. All statistical analyses were conducted in R using the *metan* package.^28^

## 3 RESULTS

### 3.1 Monitoring of stink bug populations

**Figure 1:**
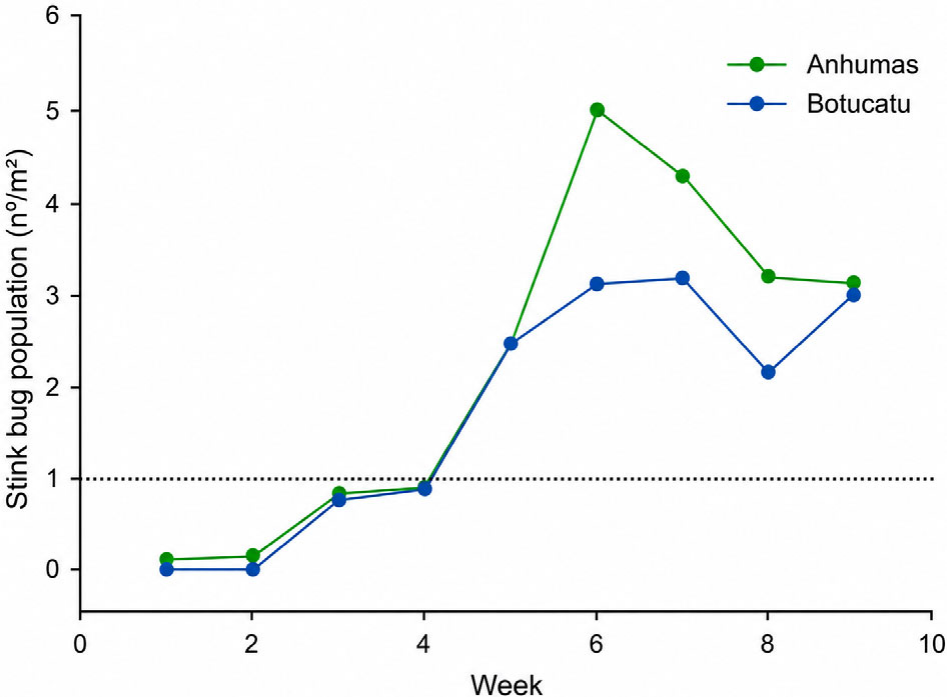
Fluctuation of mean stink bug population assessed using the beating cloth method in experiments conducted in Anhumas and Botucatu during the 2019/2020 growing season, throughout crop development. The dashed line represents the stink bug control threshold for seed production (one stink bug per square meter).

The beat cloth method revealed that the experimental fields in the Anhumas and Botucatu regions exhibited stink bug populations above the economic threshold for soybean seed production (higher than one stink bug per square meter).^19^, ^33^ In the Anhumas region, the population peaked at five stink bugs per square meter, while in Botucatu, it reached approximately three stink bugs per square meter (Fig. 1). The stink bug species detected in both regions were *Nezara viridula* (L.), *Piezodorus guildinii* (W.), *Diceraeus melacanthus* (D.), and *Euschistus heros* (F.).

### 3.2 General data overview and genotype performance for seed physiological quality traits across evaluated environments

**Figure 2:**
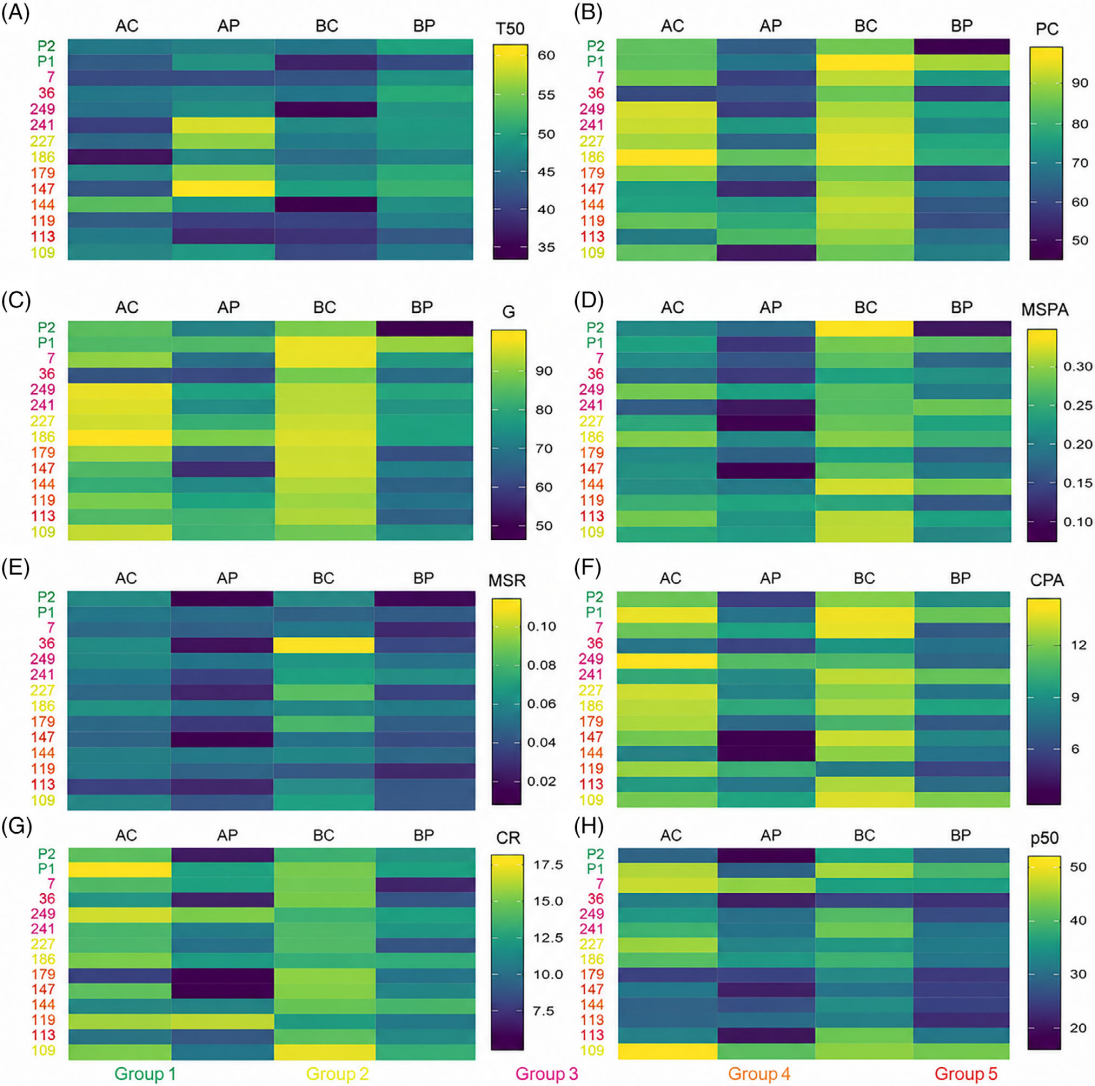
Performance heatmaps of 14 soybean genotypes (two parental lines and 12 RILs) across four evaluation environments associated with stink bug complex. (A) Time to reach 50% germination (T50). (B) First germination count at 5 days (PC). (C) Final germination (G). (D) Shoot dry mass (MSPA). (E) Root dry mass (MSR). (F) Shoot length (CPA). (G) Root length (CR). (H) Time to 50% loss of viability (p50). Genotypes were color‐coded according to their previously classified levels of seed physiological quality and tolerance to stink bug complex (Table 1). *x* axis, genotypes; *y* axis, environments; P1, IAC‐100 parental line; P2, CODETEC 215 parental line; AC, Anhumas with chemical stink bug control; AP, Anhumas without insecticide‐based control; BC, Botucatu with insecticide‐based control; BP, Botucatu without insecticide‐based control.

Fourteen genotypes (12 RILs and two contrasting parental lines) were evaluated for seed physiological traits under stink bug infestation levels across four evaluation environments, defined by two regions (Anhumas and Botucatu) and two management scenarios (with and without insecticide‐based control). Heatmap analyses highlighted differential genotype performance for seed physiological quality traits across environments (Fig. 2). Environmental effects were more pronounced for PC, G, CPA, and CR, whereas T50, MSPA, MSR, and p50 showed comparatively stronger genotypic influence alongside environmental effects. For PC, G, CPA, and CR, genotypes generally performed better under insecticide‐based control (AC and BC), particularly in BC. In contrast, AP concentrated superior genotypes for T50, BC for MSPA and MSR, and AC for p50.

Except for T50, parent IAC‐100 outperformed CD‐215 across environments (Fig. 2), consistent with its previously reported tolerance to the stink bug complex.^18^ Genotypes from Group 2 (109, 186, and 227) consistently ranked among the best performers, especially under insecticide‐based control (AC and BC). ANOVA and Fisher's LSD tests for p50 further confirmed the superior longevity of IAC‐100 and Group 2 RILs (Supporting Information, Fig. S1).

### 3.3 Joint ANOVA for all variables across environments

Based on the joint ANOVA, the proportion of variance explained by ENV, GEN, GEI, and residuals was estimated for the eight seed physiological traits (Table 2). ENV exerted a strong effect on PC, G, MSPA, and CPA, explaining 51.6%, 47.4%, 36.0%, and 39.7% of the phenotypic variation, respectively. In contrast, GEI accounted for a substantial proportion of variation in T50, MSR, and CR (28.1%, 35.7%, and 32.8%, respectively). The genotype effect was particularly pronounced for p50, explaining 53.2% of the phenotypic variation (Table 2), indicating strong genetic control of seed longevity under stink bug infestation levels. Collectively, these results highlight the predominant environmental influence on early vigor‐related traits and the stronger genetic contribution to longevity.

**Table 2:** Joint analysis of variance (ANOVA) for eight measured variables (T50, PC, G, MSPA, MSR, CPA, CR, and p50) in 14 soybean genotypes (two parental lines and 12 RILs) across four evaluation environments associated with the stink bug complex

Variable	Factor	df	Sum sq	Var %	Mean sq	*F* value
T50	ENV	3	1543.44	15.3	514.48	21.23***
	GEN	13	1926.26	19.1	148.17	6.12***
	GEN × ENV	39	2837.86	28.1	72.76	3***
	Residuals	156	3780	37.5	24.23	
	Total	211	10 087.56	100		
PC	ENV	3	24 400.82	51.6	8133.6	358.5***
	GEN	13	8351.03	17.6	642.39	28.32***
	GEN × ENV	39	11 048.61	23.3	283.3	12.49***
	Residuals	156	3539.26	7.5	22.69	
	Total	211	47 339.72	100		
G	ENV	3	17 815.91	47.4	5938.64	181.82***
	GEN	13	7376.88	19.6	567.45	17.37***
	GEN ×ENV	39	7332.34	19.5	188	5.76***
	Residuals	156	5095.07	13.5	32.66	
	Total	211	37 620.2	100		
MSPA	ENV	3	0.4	36	0.13	90.53***
	GEN	13	0.17	15.3	0.013	8.8***
	GEN × ENV	39	0.31	28	0.008	5.42***
	Residuals	156	0.23	20.7	0.0015	
	Total	211	1.11	100		
MSR	ENV	3	0.025	35.7	8.4	139.52***
	GEN	13	0.01	14.3	7.46	12.4***
	GEN × ENV	39	0.025	35.7	6.43	10.7***
	Residuals	156	0.01	14.3	6.01	
	Total	211	0.07	100		
CPA	ENV	3	834.54	39.7	278.18	137.83***
	GEN	13	381.47	18.1	29.34	14.54***
	GEN ×ENV	39	572.6	27.2	14.68	7.27***
	Residuals	156	314.85	15	2.02	
	Total	211	2103.46	100		
CR	ENV	3	672.22	26.6	224.07	61.65***
	GEN	13	459.15	18.2	35.32	9.72***
	GEN × ENV	39	828.68	32.8	21.25	5.85***
	Residuals	156	567	22.4	3.63	
	Total	211	2527.05	100		
p50	ENV	3	3159	21.8	1052.93	168.44***
	GEN	13	7720.04	53.2	593.85	95***
	GEN × ENV	39	2657.25	18.3	68.13	10.9***
	Residuals	156	975.14	6.7	6.25	
	Total	211	14 511.43	100		

Abbreviation: df, degrees of freedom; Sum sq, sum of squares; Var %, percentage of variation explained by factor; Mean sq, mean square; *F* value, *F* statistic (ANOVA; *P* < 0.05); *P < 0.001; ENV, environment; GEN, genotype; GEN × ENV: genotype‐by‐environment interaction; T50, time to reach 50% germination; PC, first germination count at 5 days; G, final germination; MSPA, shoot dry mass; MSR, root dry mass; CPA, shoot length; CR, root length; p50, time to 50% loss of viability.

### 3.4 Correlation analysis among the variables

**Figure 3:**
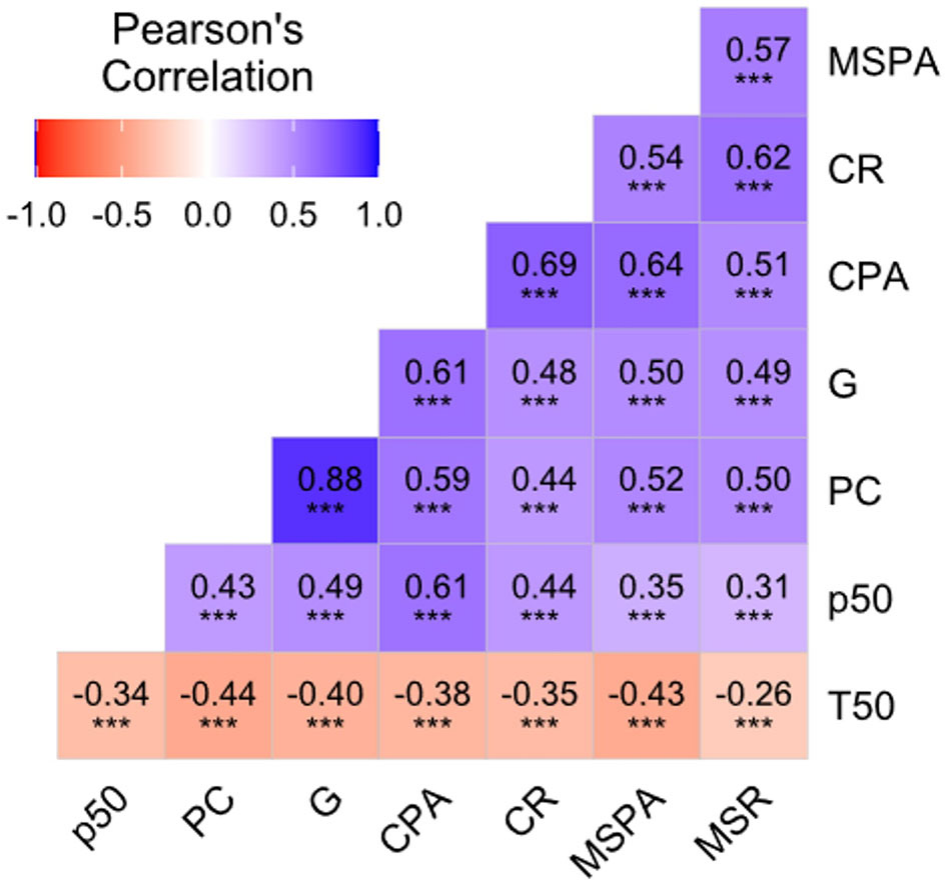
Pearson correlation matrix among all pairs of eight measured variables (T50, PC, G, MSPA, MSR, CPA, CR, and p50) in 14 soybean genotypes (two parental lines and 12 RILs) across four evaluation environments associated with stink bug complex. Positive correlations are shown in blue and negative correlations in red. Color intensity is proportional to correlation coefficient (values displayed within each box representing the pairwise interaction between variables). *P < 0.001; T50, time to reach 50% germination; PC, first germination count at 5 days; G, final germination; MSPA, shoot dry mass; MSR, root dry mass; CPA, shoot length; CR, root length; p50, time to 50% loss of viability.

The correlation heatmap (Fig. 3) showed significant associations (*P* < 0.001) among the eight seed physiological traits. Strong positive correlations were observed among PC, G, MSPA, MSR, CPA, CR, and p50, with PC and G exhibiting the highest correlation. In contrast, T50 was significantly and negatively correlated with all other variables, with correlations ranging from low (MSR) to moderate (p50, PC, G, CPA, CR, and MSPA).

### 3.5 GEN × ENV interaction

#### 3.5.1 Genotype main effects: AMMI biplots

**Figure 4:**
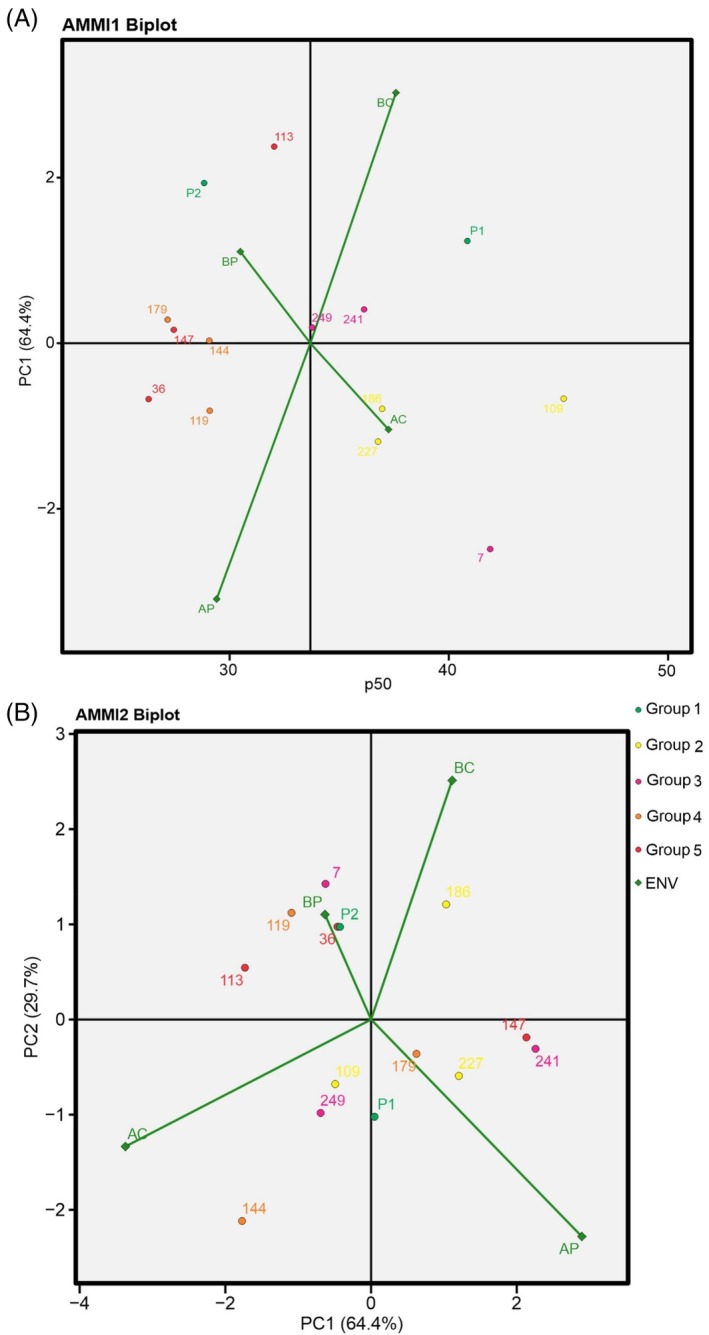
Biplots of main effects from the additive main effect and multiplicative interaction (AMMI) model indicating genotype‐by‐environment interaction for 14 soybean genotypes (two parental lines and 12 RILs) across four evaluation environments associated with the stink bug complex. (A) AMMI1 for p50 variable (*x* axis). On the *y* axis, PC1 explains 66.4% of total variation. (B) PCA of seed physiological quality data for 14 soybean genotypes across the four evaluation environments, explaining 94.1% of total variation. On the *x* axis, PC1 accounts for 64.4%, while PC2 on the *y* axis explains 29.7%. Genotypes were color‐coded according to their prior classification regarding seed physiological quality and tolerance to stink bug complex (Table 1). PCA, principal component analysis; ENV, environment; P1, IAC 100 parental line; P2, CODETEC 2015 parental line; AC, Anhumas with insecticide‐based control of stink bugs; AP, Anhumas without insecticide‐based control of stink bugs; BC, Botucatu with insecticide‐based control of stink bugs; BP, Botucatu without insecticide‐based control of stink bugs.

Because p50 (seed longevity) was the only variable for which the genotype effect explained a substantial proportion of phenotypic variation (Table 2), its adaptability and stability patterns were further explored using AMMI biplot analyses. The AMMI1 biplot (Fig. 4(A)) integrates genotype main effects and the first principal component of the multiplicative interaction (PC1), enabling simultaneous visualization of mean performance and interaction magnitude.

Genotypes 249 and 241 (group 3), located near the origin, exhibited higher stability across environments, indicating reduced GEI influence on seed longevity. In contrast, P1, 109, and 7 showed longevity values above the overall mean but were positioned farther from the origin along PC1, indicating stronger interaction with environmental conditions. Genotypes 144 and 147 presented below‐average longevity; among them, 147 was positioned closer to the origin, suggesting low responsiveness to environmental variation despite reduced performance (Fig. 4(A)).

Regarding environmental effects, AC and BP showed lower PC1 scores and therefore contributed less to GEI, whereas AP and BC were positioned farther from the origin, indicating stronger interaction effects. Notably, AP and BC showed strong PC1 magnitudes in the AMMI1 biplot (Fig. 4(A)), indicating stronger interaction effects for seed longevity. Although both environments contributed substantially to GEI, the more pronounced differences among genotypes observed under AP conditions (Supporting Information, Fig. S1) reinforce its relevance as a selective environment.

The AMMI2 biplot (Fig. 4(B)), incorporating PC1 and PC2, provided additional resolution of interaction patterns. BP and BC were positioned closer together in the biplot, suggesting related interaction responses despite differences in chemical management. In contrast, AP showed a strong interaction magnitude considering PC1 and PC2 scores together (Supporting Information, Table S1), highlighting its relevant contribution to GEI for p50. Genotypes 144, 113, 147, and 241 displayed limited interaction magnitude, whereas P1, 109, and 249 showed comparable interactive behavior across environments, particularly under AC and AP conditions. Specific environmental adaptation was observed for P2, 7, 36, and 119 toward BP, and for genotype 186 toward BC, as indicated by their proximity to the respective environmental vectors. The numerical scores underlying the AMMI2 biplot for p50 are provided in Supporting Information, Table S1, supporting the interpretation of mean performance, distance from the origin, and GEI patterns.

#### 3.5.2 Selection of the most informative genotype and environment: GGE biplots

**Figure 5:**
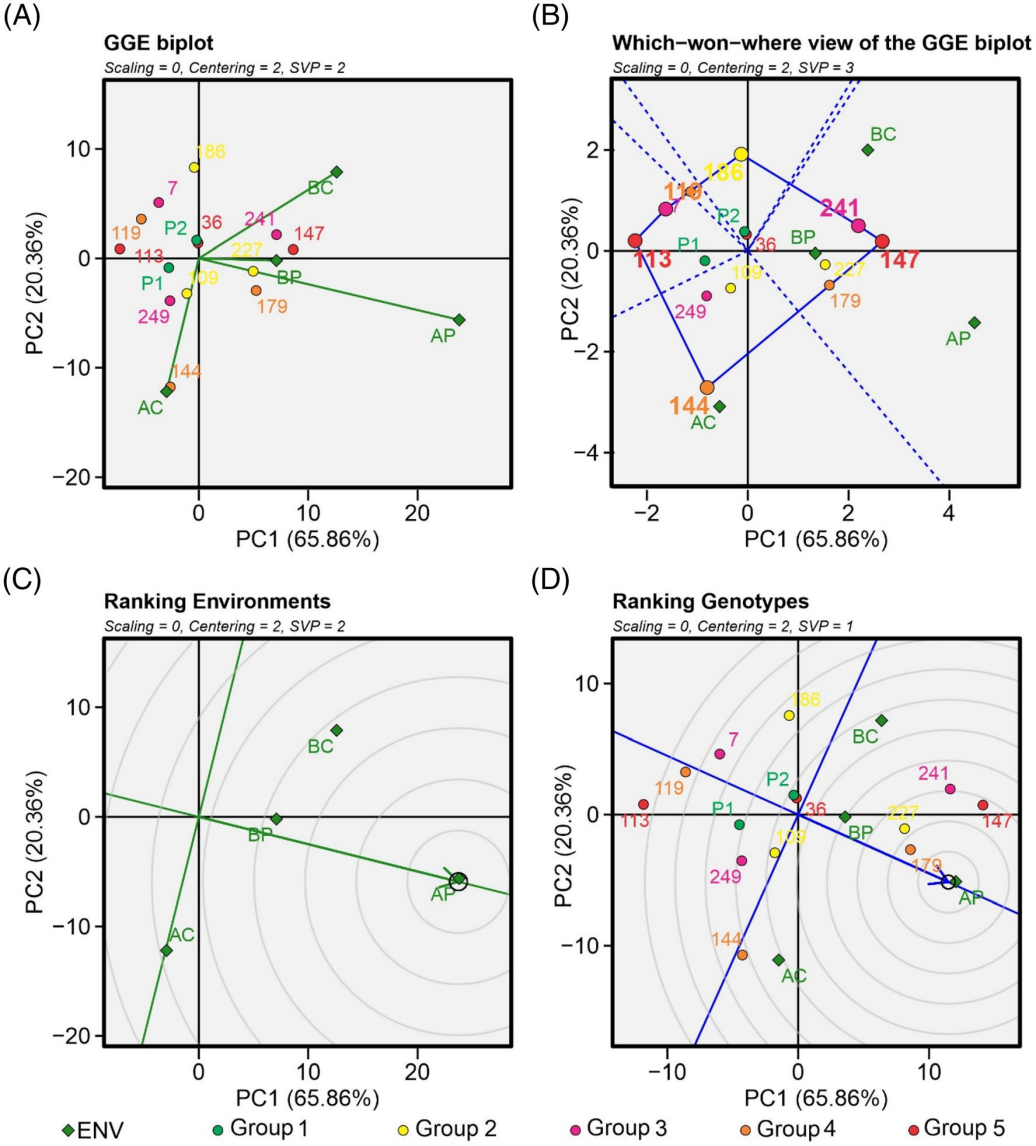
Biplots of main effects from the genotype plus genotype‐by‐environment interaction (GGE) model indicating multiple interactions for 14 soybean genotypes (two parental lines and 12 RILs) across four evaluation environments associated with the stink bug complex. (A) GGE biplot analysis showing a PCA for representation and discrimination of genotypes across the evaluated environments. (B) GGE biplot analysis in ‘which‐won‐where’ view, indicating a polygon within the PCA used to identify the best‐performing genotypes across the tested environments. (C) GGE biplot analysis focusing on environments, establishing a ranking among them and indicating the most ideal environment (closest to central point of concentric circles) for evaluating seed physiological quality and tolerance to stink bug in this population. (D) GGE biplot analysis presenting a PCA‐based ranking of genotypes, where proximity to central point of concentric circles reflects better performance across environments, based on mean performance and stability. Genotypes were color‐coded according to their prior classification regarding seed physiological quality and tolerance to stink bug complex (Table 1). PCA, principal component analysis; ENV, environment; P1, IAC 100 parental line; P2, CODETEC 2015 parental line; AC, Anhumas with insecticide‐based control of stink bugs; AP, Anhumas without insecticide‐based control of stink bugs; BC, Botucatu with insecticide‐based control of stink bugs; BP, Botucatu without insecticide‐based control of stink bugs.

GGE biplot analyses were performed to further dissect GEI patterns across the eight seed physiological traits (Fig. 5). The environment‐focused biplot (Fig. 5(A)) revealed differences in discriminative ability and representativeness among the four evaluation environments. AP showed the longest vector length, indicating again its discriminative capacity, whereas AC, BC, and BP exhibited shorter vectors and therefore contributed less to genotype differentiation. BP was positioned closer to the average environment axis, indicating higher representativeness, while AP combined strong discriminative power with practical relevance for genotype selection under stink bug infestation levels.

The ‘which‐won‐where’ polygon view (Fig. 5(B)) enabled identification of mega‐environments based on genotype performance sectors. Two mega‐environments were detected: ME1 (AP, BC, and BP) and ME2 (AC). Genotypes located at the vertices of each sector were considered specifically adapted. Genotypes 109, 249, and 144 were associated with superior performance in ME1, whereas 241, 147, 227, and 179 showed better responsiveness within ME2. Genotypes positioned within interior sectors displayed lower specificity of adaptation.

The ideal‐environment ranking (Fig. 5(C)), based on concentric circles centered on the average environment coordinate, confirmed AP as the most informative and desirable testing environment, while AC showed the lowest selection efficiency. Finally, the genotype‐ranking view (Fig. 5(D)) identified genotype 179 as the closest to the ideal genotype, combining stability and mean performance, followed by 227, which exhibited higher mean performance but slightly lower stability. Conversely, genotype 113 was the most distant from the ideal position, reflecting lower overall performance and stability. Notably, genotype 179 is tolerant to the stink bug complex, whereas 113 is susceptible (Table 1), reinforcing the alignment between physiological performance and pest tolerance.

#### 3.5.3 Estimates of genetic parameters and genotype plots based on the BLUP model

Only p50 showed a significant genotypic effect in the BLUP analysis (Table 3), consistent with the joint ANOVA results (Table 2), in which seed longevity was the only trait predominantly controlled by genotype. In contrast, the GEN × ENV interaction was highly significant for all variables, confirming differential genotype responses across AC, AP, BC, and BP environments and reinforcing the relevance of GEI modeling.^32^

**Table 3:** Estimates of variance components and genetic parameters based on a linear mixed model using the BLUP method for eight measured variables (T50, PC, G, MSPA, MSR, CPA, CR, and p50) in 14 soybean genotypes (two parental lines and 12 RILs) across four evaluation environments associated with the stink bug complex

	T50	PC	G	MSPA	MSR	CPA	CR	p50
V_G_	4.71^ns^	22.44^ns^	23.72^ns^	0.00031^ns^	0.0000064^ns^	0.92^ns^	0.88^ns^	32.86***
V_GEI_	12.13***	65.15***	38.84***	0.0016***	0.00015***	3.17***	4.4***	15.47***
V_R_	24.23	22.69	32.66	0.0015	0.00006	2.02	3.63	6.25
*H*	0.11	0.2	0.25	0.09	0.03	0.15	0.1	0.6
*R* ^2^_GEI_	0.3	0.59	0.41	0.48	0.69	0.52	0.49	0.28
Ac	0.71	0.75	0.82	0.62	0.37	0.71	0.63	0.94
*r*_ge_	0.33	0.74	0.54	0.52	0.71	0.61	0.55	0.71

Abbreviation: VG, genotypic variance; VGEI, genotype‐by‐environment interaction variance; VR, residual variance; *H*, broad‐sense heritability; *R* ^2^_GEI_, coefficient of determination for genotype‐by‐environment interaction; Ac, selective accuracy; *r*_GE_, genotype‐environment correlation; ns, not significant (likelihood ratio test; *P* < 0.05); *P < 0.001; T50, time to reach 50% germination; PC, first germination count at 5 days; G, final germination; MSPA, shoot dry mass; MSR, root dry mass; CPA, shoot length; CR, root length; p50, time to 50% loss of viability.

Among the evaluated traits, p50 stood out as the one under strongest genetic control, as indicated by its *H* value (0.6) and relatively lower contribution of GEI (R^2^GEI = 0.28) (Table 3). In contrast, most vigor‐related traits showed lower heritability and higher R^2^GEI values, indicating stronger environmental influence on their expression. The Ac for p50 was the highest (0.94), supporting the reliability of the predicted genotypic values. Already the rge values ranged from 0.33 (T50) to 0.74 (PC) across traits, reinforcing the relevance of GEI information for selection decisions.

**Figure 6:**
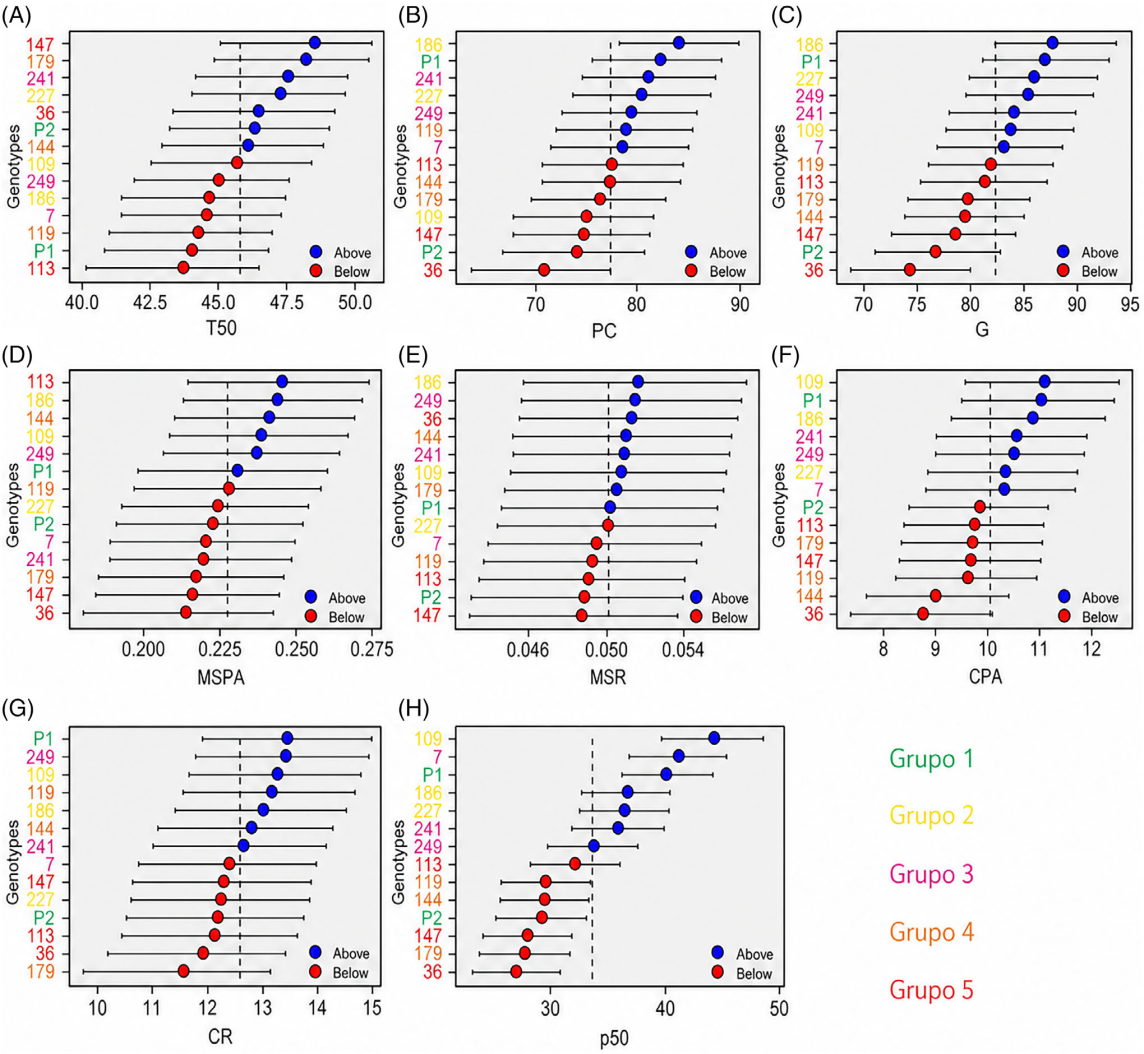
Best predicted mean values from the best linear unbiased prediction (BLUP) for eight measured variables in 14 soybean genotypes (two parental lines and 12 RILs). (A) Time to reach 50% germination (T50). (B) First germination count at 5 days (PC). (C) Final germination percentage (G). (D) Shoot dry mass (MSPA). (E) Root dry mass (MSR). (F) Shoot length (CPA). (G) Root length (CR). (H) Time to 50% loss of viability (p50). Genotypes were color‐coded according to their prior classification based on seed physiological quality and tolerance to stink bug complex (Table 1). P1, IAC 100 parental line; P2, CODETEC 2015 parental line.

Predicted genotypic means across environments (Fig. 6) further highlighted p50 as the most genetically consistent trait. Genotypes 109 and 7 showed superior predicted means for p50, while 186 and P1 (IAC‐100) ranked superior for PC and G. For CPA and CR, superior predicted means were observed for 109, P1, and 249. Regarding MSPA and MSR, 186 and 249 consistently ranked among the best‐performing genotypes. Notably, RILs from Group 2 (109, 186, and 227) and the tolerant parent IAC‐100 were repeatedly positioned among the top predicted means across multiple traits, including seed longevity. This pattern corroborates the higher proportion of genotypic variance explained for p50 (53.2%; Table 2), its higher heritability (*H* = 0.6), and higher selective accuracy (Ac = 0.94) (Table 3), reinforcing longevity as a robust genetic target under stink bug infestation levels.

#### 3.5.4 MTSI analysis, predicted selection differential, and TOP1 genotypes per environment

**Figure 7:**
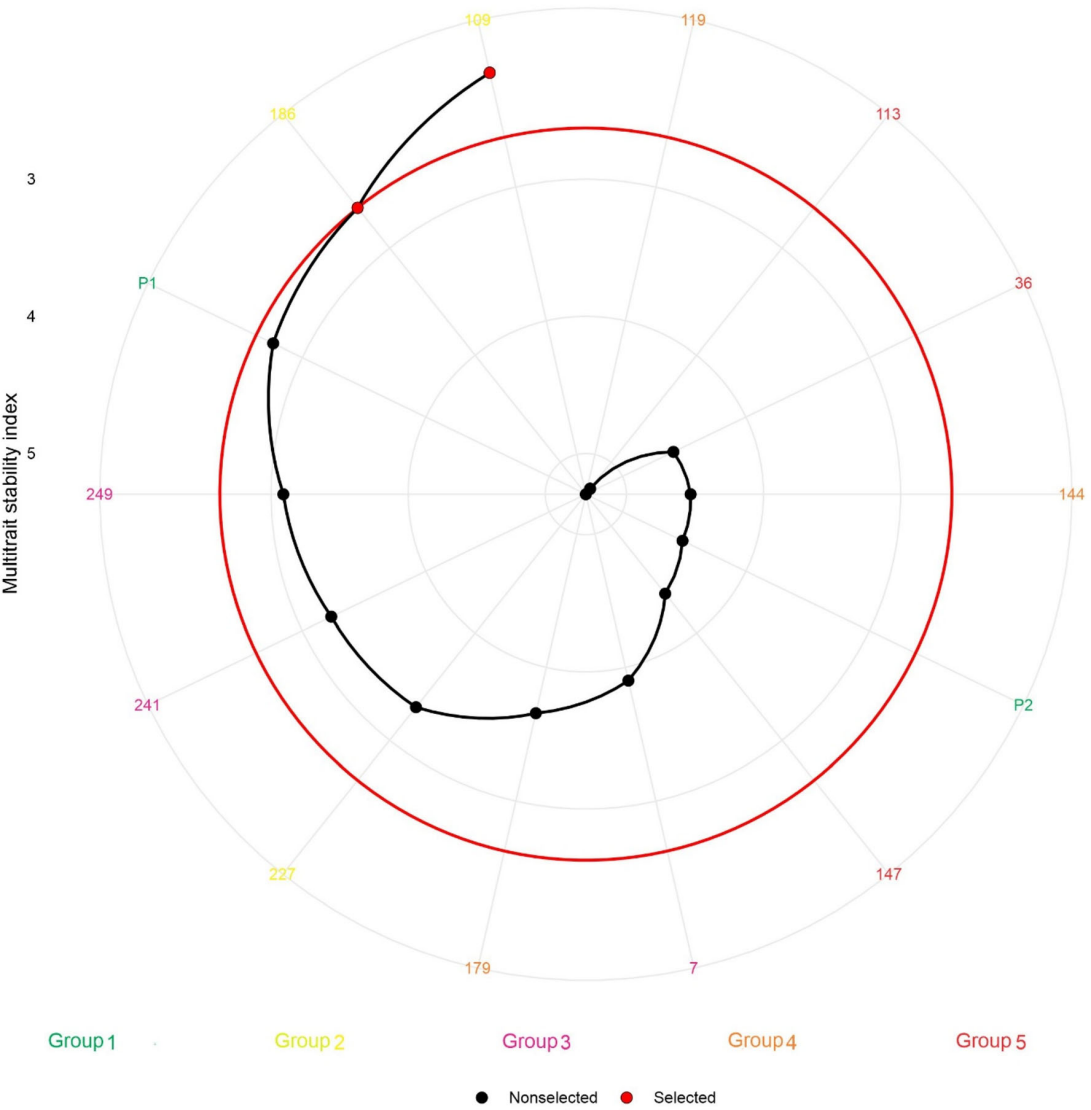
Ranking of 14 soybean genotypes (two parental lines and 12 RILs) in ascending order based on MTSI index. Genotypes were color‐coded according to their prior classification regarding seed physiological quality and tolerance to stink bug complex (Table 1). MTSI, Multi‐Trait Stability Index; P1, IAC 100 parental line; P2, CODETEC 2015 parental line.

The MTSI was applied to rank 14 genotypes across eight seed physiological traits evaluated in four evaluation environments. Under a 15% selection intensity, genotypes 109 and 186 showed the lowest MTSI values, indicating superior mean performance combined with strong stability (Fig. 7). Both belong to Group 2, previously characterized by high physiological seed quality and tolerance to the stink bug complex (Table 1), reinforcing the consistency of multi‐environment selection.

Selection differential estimates derived from the MTSI (Table 4) indicated positive gains for all variables except T50, which showed a negative selection differential. This pattern is coherent with the negative correlation of T50 with the remaining traits (Fig. 3), since lower T50 values indicate higher vigor. Among all traits, p50 exhibited the highest broad‐sense heritability (*H* ^2^ = 0.89), consistent with previous variance component estimates (Tables 2 and 3), further supporting seed longevity as a genetically robust selection target.

**Table 4:** Predicted selection differential for eight measured variables (T50, PC, G, MSPA, MSR, CPA, CR, and p50) based on the MTSI index

	T50	PC	G	MSPA	MSR	CPA	CR	p50
Factor 1	−0.67	−0.3	−0.39	−0.31	−0.84	−0.16	−0.73	−0.92
Gain	Increase	Increase	Increase	Increase	Increase	Increase	Increase	Increase
*X*_0_	45.71	77.46	82.31	0.22	0.05	10.04	12.34	33.68
*X*_s_	44.35	80.63	87.94	0.26	0.06	11.75	13.67	41.1
SD	−1.36	3.16	5.63	0.03	0.01	1.71	1.32	7.41
SD %	−2.97	4.08	6.83	15.09	12.93	17.02	10.72	22.01
*H* ^2^	0.51	0.56	0.67	0.38	0.14	0.5	0.4	0.89

Abbreviation: MTSI, Multi‐Trait Stability Index; *X*₀, original value; *X*_s_, selected value; SD, selection differential; SD %, selection differential in percentage; *H* ^2^, broad‐sense heritability; T50, time to reach 50% germination; PC, first germination count at 5 days; G, final germination percentage; MSPA, shoot dry mass; MSR, root dry mass; CPA, shoot length; CR, root length; p50, time to 50% loss of viability.

The TOP1 analysis across environments (Table 5) revealed that genotype 147 ranked highest for T50 in most environments (AP, BC, and BP), reflecting lower vigor and aligning with its classification in Group 5 (low physiological quality and susceptibility) (Table 1). In contrast, P1 (IAC‐100), 109, and 186 consistently ranked among the best for the remaining seven physiological traits across environments, confirming their superior performance and stability under stink bug infestation levels (Table 1).

**Table 5:** The top 1 ranking based on MTSI index for 14 soybean genotypes (two parental lines and 12 RILs) across the four evaluation environments associated with the stink bug complex

Trait	AC	AP	BC	BP
T50	144	147	147	147
PC	186	186	P1	P1
G	186	186	7	P1
MSPA	186	119	P2	144
MSR	186	144	36	241
CPA	249	249	P1	109
CR	P1	119	109	144
p50	109	7	P1	109

Abbreviation: Genotypes were color‐coded according to their prior classification regarding seed physiological quality and tolerance to stink bug complex (Table 1). MTSI, Multi‐Trait Stability Index; P1, IAC 100 parental line; P2, CODETEC 2015 parental line; AC, Anhumas with insecticide‐based control of stink bugs; AP, Anhumas without insecticide‐based control of stink bugs; BC, Botucatu with insecticide‐based control of stink bugs; BP, Botucatu without insecticide‐based control of stink bugs.

## 4 DISCUSSION

Many plant‐breeding programs prioritize high yield and tolerance to biotic and abiotic stresses; however, limited attention has been given to how seed physiological quality behaves under pest exposure within a GEI framework. To date, no studies have reported GEI analyses in segregating soybean populations incorporating seed physiological traits as selection criteria under stink bug infestation in the maternal plant, a major tropical pest frequently associated with qualitative losses in soybean grains and seeds. In seed production systems, stink bug injury translates into economic losses not only via yield but also via reductions in seed marketability and stand establishment, making seed physiological stability an IPM‐relevant outcome.

Previous studies have primarily focused on genetic parameters for germination and vigor in segregating populations^34^, ^35^, ^36^ or on the physiological and biochemical responses of seeds to stink bug herbivory.^4^, ^37^, ^38^ These investigations demonstrate that developing seeds activate rapid defensive strategies, including earlier maturation, detection of punctures within minutes, and induction of defense‐related genes and metabolic pathways that alter insect feeding behavior by reducing appetite and impairing digestive enzyme accumulation.^4^, ^37^, ^38^ From a pest management perspective, such mechanisms suggest that host plant traits can directly influence pest performance, reinforcing the role of genetic tolerance as a cornerstone of sustainable control strategies.

Prioritizing selection based on seed physiological traits at advanced breeding stages, particularly under stink bug exposure, may therefore represent an efficient pathway for developing cultivars capable of maintaining performance in pest‐prone environments. The higher broad‐sense heritability estimates observed for these variables support their usefulness as management‐relevant traits and indicate that seed traits can serve as reliable proxies for tolerance. Because these traits are shaped by multiple genes and environmental drivers, integrating GEI into breeding pipelines becomes essential for identifying genotypes capable of buffering pest‐induced stress.^39^

All eight seed physiological variables evaluated here were influenced by GEI across the four evaluation environments, although the magnitude varied among traits. Variables such as PC, G, CPA, and CR were more strongly affected by environmental conditions, indicating higher sensitivity to production scenarios, whereas T50, MSPA, MSR, and especially p50 exhibited higher stability, suggesting stronger genetic control (Fig. 2). This pattern highlights two important biological processes: the plasticity of viability‐related traits in response to environmental fluctuation and the genetic determination of vigor‐ and lifespan‐related attributes. Although physiological quality parameters often display reduced stability across environments,^34^ our findings indicate that some traits remain genotype‐driven even under pest exposure, emphasizing the importance of trait‐specific analyses when designing breeding strategies.^40^

Notably, longevity (p50) showed a substantial proportion of phenotypic variation explained by genotype (Table 2). Seed storability has gained relevance not only for breeding but also for production logistics, as it reduces viability loss during storage and correlates strongly with germination and vigor.^41^ From a management standpoint, cultivars capable of maintaining seed longevity despite stink bug attack may contribute to more resilient production systems, particularly in regions where pest outbreaks coincide with unfavorable climatic conditions. Additionally, seeds with low viability are naturally eliminated during breeding cycles, reinforcing longevity as a practical and indirectly selectable trait.

Correlation analyses further supported the integration of physiological traits into selection frameworks. The strong positive association between germination (G) and PC indicates that vigor and seedling normality respond similarly over time, whereas moderate correlations among the remaining variables suggest complementary contributions to seed performance (Fig. 3). Germination, when combined with vigor tests, remains a reliable parameter for genotype selection and genetic gain.^35^ As expected, T50 was negatively correlated with other variables and continues to serve as a widely accepted vigor indicator representing the time required for 50% germination.^23^, ^42^ Identifying these relationships enables indirect selection of complex traits through simpler measurements, improving efficiency in breeding programs.^43^

The AMMI model proved valuable for identifying stability patterns and environmental influences on GEI.^44^ Genotype 249 exhibited strong stability for longevity despite susceptibility to stink bug infestation (Table 1), whereas tolerant genotypes such as P1 and 109 (Table 1) were more responsive to environmental variation. Conversely, genotypes with low physiological quality (Table 1) showed minimal responsiveness, suggesting limited adaptive potential. Importantly, the AP environment contributed most strongly to GEI and was associated with extended seed longevity (Fig. 4), indicating that environments with higher pest infestation levels may enhance the discrimination of tolerant genotypes.

Combining AMMI with the GGE biplot allowed clearer identification of mega‐environments and selection zones.^45^ The AP environment emerged as the most discriminative and therefore the most informative for selection (Fig. 5), likely reflecting the harsher production conditions in Anhumas, including elevated temperatures and higher stink bug populations. High temperatures during seed development are known to reduce germination and increase pathogen incidence, lowering seed market value.^46^ Simultaneously, declining efficacy of insecticides due to resistance has been documented in stink bug populations attacking soybean and other major crops in South America.^47^, ^48^ Together, these factors reinforce the importance of host plant tolerance as part of IPM.

The contrast between chemical and non‐chemical scenarios further supports this interpretation. The no‐insecticide treatment in Anhumas generated stronger selection pressure and provided more informative discrimination among genotypes, whereas insecticide‐based control appeared to buffer environmental stress (Figs 4 and 5). Although seed quality was lower under AP conditions, the increased selection pressure improved the detection of superior materials, suggesting that breeding under realistic pest scenarios may accelerate genetic gain, with AP being most informative for IPM‐relevant validation under infestation. The GGE analyses confirmed that genotypes differed in adaptability across mega‐environments, enabling simultaneous selection for performance and stability (Fig. 5).^16^, ^45^, ^49^ Such approaches have been successfully applied to several crops^49^, ^50^, ^51^ and are particularly valuable when targeting resilience under adverse conditions. Already the BLUP methodology provided accurate estimates of genotype performance^52^ and reinforced longevity as a genetically driven trait with moderate GEI influence. Similar benefits of BLUP‐based selection have been reported across multiple crops.^17^, ^53^, ^54^, ^55^ Seed longevity is known to depend on structural and environmental factors, including phenology and storage conditions (Zani and Müller, 2017; Guzzon *et al*., 2021),^56^, ^57^ and our findings expand this understanding by demonstrating a strong genetic contribution under stink bug infestation levels.

Integrating AMMI and BLUP through WAASB enabled simultaneous evaluation of performance and stability,^32^ highlighting Group 2 RILs (109, 186, 227) and parent IAC‐100 as superior materials (Fig. 6 and Table 5). This result suggests that combining high physiological quality with pest tolerance enhances performance across environments, resulting in an outcome directly aligned with sustainable pest management goals. Similarly, the MTSI approach effectively identified stable, high‐performing genotypes.^17^, ^58^, ^59^ Genotypes 109 and 186 consistently ranked among the best, while IAC‐100 demonstrated higher stability than CD‐215, indicating strong parental contrast (Fig. 7). These materials therefore represent promising candidates for breeding cultivars capable of producing seeds tolerant to stink bug attack and sustaining field establishment under infestation. This helps prioritize genotypes for deployment in high‐infestation areas.

The higher heritability observed for longevity using both MTSI and BLUP (Tables 3 and 4, respectively) reinforces its polygenic basis and links to antioxidant defenses, protein repair mechanisms, and seed coat development.^60^ Because longevity influences emergence, seedling establishment, photosynthetic capacity, and seed composition, selecting for this trait may generate cascading agronomic benefits.^61^ Understanding these mechanisms is essential for maintaining active germplasm banks and improving breeding efficiency.^62^

Ultimately, tolerance to stink bugs has the potential to generate substantial improvements across the soybean production chain. Beyond reducing agrochemical reliance, tolerant cultivars can preserve grain and seed quality, maintain product value, and support high‐performance seeds even under infestation. By reducing dependence on insecticides to maintain seed physiological quality, tolerant cultivars can also support resistance management by lowering selection pressure on stink bug populations within IPM programs. Collectively, our findings demonstrate the sensitivity of soybean seed physiological traits to stink bug injury, particularly regarding vigor and longevity. Because malformed seeds were excluded after harvest, the observed reductions are best explained by insect damage rather than purely genotypic variation. Taken together, these results position host plant tolerance as a critical component of IPM strategies. Incorporating seed physiological traits into GEI‐based selection frameworks may accelerate the development of resilient cultivars capable of sustaining productivity under increasing pest exposure. Therefore, this approach may help reduce insecticide reliance in tropical soybean systems.

## 5 CONCLUSION

This study demonstrated that GEIs play a decisive role in shaping soybean seed physiological quality under stink bug exposure. By evaluating two contrasting parents and recombinant inbred lines across four production scenarios differing in region and insecticide‐based control, we showed that host plant‐mediated mitigation of stink bug‐associated damage is strongly modulated by genetic background and environmental conditions. The Anhumas environment without insecticide‐based control (AP) functioned as a worst‐case, no‐spray validation scenario, providing the highest discriminatory power for identifying tolerant materials. We therefore recommend the inclusion of AP‐like high‐infestation, non‐insecticide environments as recurrent validation steps for IPM‐oriented selection, enabling robust discrimination between susceptible and tolerant entries. Across analyses, RILs 109, 186, and 227, together with genotypes 249 and 179 and the parental line IAC‐100 consistently maintained superior performance and represent strong candidates for advancement and IPM‐oriented deployment as sources of host plant tolerance. Among evaluated traits, seed longevity showed the highest heritability, supporting its adoption as a primary management‐relevant selection criterion under stink bug infestation levels. Collectively, these findings demonstrate that seed physiological quality should be incorporated into GEI‐informed selection pipelines as a functional component of IPM. The deployment of stable, tolerant genotypes identified under AP‐like conditions offers a practical pathway to reduce insecticide reliance while maintaining seed quality and crop establishment in tropical soybean systems.

## CONFLICT OF INTEREST

The authors declare that they have no relevant financial or personal relationships to influence what was reported in this study.

## AUTHOR CONTRIBUTIONS

LC and EAAS were involved in the conception and design of the study. LC, GFS, AAM, TBB, and SMP collected the phenotypic data. JBP provided the phenotypic data. RPML performed data analysis and interpretation. LC and RPML wrote the manuscript original draft. JBP and EAAS were responsible for funding acquisition. RPML and EAAS performed project supervision. All authors reviewed and approved the manuscript.

## Supporting information


**Figure S1.** Time in days to 50% loss of viability (p50) for the two contrasting parents and four distinct groups of RILs across evaluated environments. Bars show mean ± SE values for p50 longevity. Letters above bars indicate differences analyzed by Fisher's LSD test within each environment (*P* < 0.05). Bar colors correspond to previous classification of seed physiological quality and tolerance to stink bug complex (Table 1). P1, IAC 100 parental line; P2, CODETEC 2015 parental line; G2, Group 2; G3, Group 3; G4, Group 4; G5, Group 5.
**Table S1.** Mean p50 values and AMMI interaction scores (PC1 and PC2) for genotypes and environments underlying the AMMI2 biplot.

## Data Availability

The data that support the findings of this study are available from the corresponding author upon reasonable request.
