# Supplementary material for: Genotype‐by‐environment interaction informs selection for seed physiological quality and stink bug tolerance in soybean under pest management contrasts
Source: Pest Manag Sci. 2026 May 11;82(9):8530–44. doi: 10.1002/ps.70904 (PMC13453180; doi:10.1002/ps.70904)
Supplement: Supplementary file 1 [file PS-82-8530-s001.docx]

**SUPPLEMENTARY FIG. CAPTIONS**

**Supplementary Figure S1.** Time in days to 50% loss of viability (p50) for the two contrasting parents and four distinct groups of RILs across evaluated environments. Bars show mean ± SE values for p50 longevity. Letters above bars indicate differences analyzed by Fisher’s LSD test within each environment (p < 0.05). Bar colors correspond to previous classification of seed physiological quality and tolerance to stink bug complex (Table 1). P1 = IAC 100 parental line; P2 = CODETEC 2015 parental line; G2 = Group 2; G3 = Group 3; G4 = Group 4; G5 = Group 5.

**SUPPLEMENTARY TABLE CAPTIONS**

**Supplementary Table S1.** Mean p50 values and AMMI interaction scores (PC1 and PC2) for genotypes and environments underlying the AMMI2 biplot.

| **Factor** | **Genotype/Environment** | **Mean p50** | **PC1** | **PC2** |
| --- | --- | --- | --- | --- |
| GEN | 109 | 45.38788 | -0.49241 | -0.67859 |
| GEN | 113 | 41.49406 | -1.73166 | 0.54441 |
| GEN | 119 | 42.43656 | -1.09258 | 1.121171 |
| GEN | 144 | 46.03794 | -1.77142 | -2.11714 |
| GEN | 147 | 50.75969 | 2.131264 | -0.18848 |
| GEN | 179 | 50.17931 | 0.623204 | -0.36069 |
| GEN | 186 | 43.30731 | 1.02924 | 1.209349 |
| GEN | 227 | 48.5455 | 1.20454 | -0.59272 |
| GEN | 241 | 48.7265 | 2.256846 | -0.3079 |
| GEN | 249 | 43.91856 | -0.69205 | -0.98202 |
| GEN | 36 | 46.87463 | -0.46158 | 0.975282 |
| GEN | 7 | 43.28938 | -0.62532 | 1.425279 |
| GEN | P1 | 42.25 | 0.048264 | -1.02146 |
| GEN | P2 | 46.66269 | -0.42634 | 0.973505 |
| ENV | AC | 44.1177 | -3.372 | -1.33474 |
| ENV | AP | 48.92221 | 2.892567 | -2.28039 |
| ENV | BC | 42.30518 | 1.11204 | 2.511903 |
| ENV | BP | 47.47491 | -0.63261 | 1.103228 |
